# African American participation in cancer clinical trials

**DOI:** 10.3332/ecancer.2021.1307

**Published:** 2021-10-25

**Authors:** Jordan Swaby, Ernie Kaninjing, Motolani Ogunsanya

**Affiliations:** 1 Department of Health Sciences, University of Central Florida, 4000 Central Florida Blvd, Orlando, FL 32816, USA; 2 Department of Health Sciences, School of Health & Human Performance, Georgia College and State University, 231 W Hancock St, Milledgeville, GA 31061, USA; 3 Department of Pharmacy, Clinical & Administrative Sciences, The University of Oklahoma Health Sciences Center, 1110 N. Stonewall Ave. Oklahoma City, OK 73117, USA

**Keywords:** cancer, clinical trials, African American, minority

## Abstract

**Background:**

According to the Food and Drug Administration, African Americans (AAs) have been habitually underrepresented in cancer clinical trials (CCTs). This under-enrolment has contributed to cancer disparities despite the implementation of policies to improve AA accrual. This systematic review aimed to determine (1) Why AAs are participating in CCT at lower rates compared to other ethnic/racial groups and (2) Are there any tools that have definitively improved AA participation or addressed the barriers associated with their lack of participation.

**Methods:**

Searches were carried out in PubMed, Project MUSE and EBSCO which were confined to four databases (BASE, PsycINFO, CINAHL and MEDLINE). Literature published between 2010 and 2020 were filtered with the inclusion and exclusion criteria and then a mixed methods appraisal tool was used to check the quality of the articles. Studies were separated into two categories to extract and synthesise data based on the emerging themes.

**Results:**

Frequent reasons for a lack of participation involved provider related issues, family concerns, health literacy and trust among others. Interventions cited as successful in improving AA participation or addressing a barrier often revolved around community-based participatory research and educational CCT videos/tools.

**Recommendations/Conclusion:**

Educating AA patients about the biomedical research process, addressing concerns about CCTs, building trust with community members and improving communication with healthcare providers could improve AA participation in CCTs. Future interventions should consider the effect of diversified healthcare teams in addressing trust deficit in CCTs among AAs. Healthcare practitioners seeking to consent AA into CCTs and biomedical research could consider incorporating cultural competence into their practice for effective interaction with this population and to address their questions about biomedical research.

## Introduction

In 2019, the Food and Drug Administration issued guidance to broaden the eligibility criteria for clinical trials to include minorities who have been habitually underrepresented [1]. Minorities, according to the National Institutes of Health (NIH) are Asians, African Americans (AAs), Native Americans, Pacific Islanders and Hispanics [2]. Of these minorities, Hispanics and AA are among the largest groups and experience some of the highest cancer health disparities in the nation with AA experiencing a higher disease burden [35]. This guidance aimed to not only increase minority participation in clinical trials but also make it easier for the researchers to develop an approach to do so, thereby combating health disparities. The Centers for Disease Control and Prevention (CDC) defines health disparities as preventable variances in the burden of disease that are typically experienced by socially disadvantaged people [3]. Unequal distribution of disease burden within ethnic groups can decrease quality of life and increase mortality rate, which demonstrates the need to address health disparities. Although other government agencies such as the NIH have implemented policies to improve minority participation in cancer clinical trials (CCTs) [2], AA participation remains low at 5% [27].

In the United States, AA experiences some of the highest mortality rates for breast, prostate and lung cancer [4–6]. Between 2003 and 2012, breast cancer mortality decreased in White women in all 50 states as opposed to AA women, which only saw a decrease in 27 of the 30 states for which data was available [5]. As of 2015, AA and White men’s prostate cancer incidence differed substantially with AA men experiencing an incidence rate of 178.3 per 100,000 compared to their white counterparts with 105.7 per 100,000 men [6]. Unfortunately, not only is the incidence rate higher, but the late-stage diagnosis is between 44% and 75% higher in AA males [7] in contrast to the general public. According to the CDC, in 2017, lung cancer incidence rates in AA and White men also exhibited a disparity with 71.7 new cases per 100,000 for AA men compared to 62.6 per 100,000 for White men [8]. Reasons for these disparities include low socioeconomic status, poor physician–patient communication and low participation in CCTs [9–11].

CCTs which include testing of novel therapies have the potential to lead to new cancer treatments. Properly designed CCT with a representative sample of participants (age, gender, race and geographic location) can yield findings generalisable to the entire population [16]. Studies conducted with minimal or no participation of AA or other minority groups face the challenge of generalising their findings to the entire population. To ensure that CCT are conducted among a diverse and representative population, it is paramount that researchers understand the determinants of low CCT participation rates in AAs relative to the higher rates observed in other races/ethnicities. This understanding can lead to the development of interventions that improving health equity and better outcomes among this subpopulation. To explore and better understand the lower CCT participation rate among AA, a systematic review was conducted. The research questions for this systematic review were (1) Why do AA participates in CCT at lower rates compared to other ethnic/racial groups and (2) Are there examples of definitive tools that have helped increase AA participation or addressed barriers associated with their lower participation rate?

## Methods

To identify relevant articles related to this topic, a search was conducted in June 2020 using the following databases: PubMed, CINAHL, BASE, PsycINFO, Medline and Project MUSE. EBSCO was used solely to search PsycINFO, BASE, CINAHL and MEDLINE simultaneously. Inclusion criteria were studies on cancer, clinical trials, AA and minority. The scope of the search was limited to articles published between 2010 and 2020. Exclusion criteria were studies focused on screening, quantitative studies geared towards quantifying the participation rates in clinical trials, narrative and systematic reviews.

The search terms, African American participation in cancer clinical trials, yielded a total of 477 articles. Of the 477 articles, 165 were generated from PubMed, 175 from EBSCO and 137 from Project MUSE. The first round of screening involved simply removing duplicate articles present in multiple databases, leading to a total of 389 unique articles.

The second round of screening entailed examining the article titles and abstracts to determine if they met the inclusion criteria. Articles that met four out of the five inclusion criteria were first examined by reviewing their objectives, results and conclusions from the abstract to determine their applicability to the research questions. For example, if an article was published from 2010 to 2020 and included cancer, clinical trials and minorities but not AA, the abstracts were examined to determine if the results section provided distinct data for AA. The third round of screening filtered articles that were not capable of answering the research questions. Articles that did not have a sample population containing at least 14% AA were excluded to ensure the studies had proper representation that mirrored the proportion of AA population in the United States [12].

**Figure 1.**
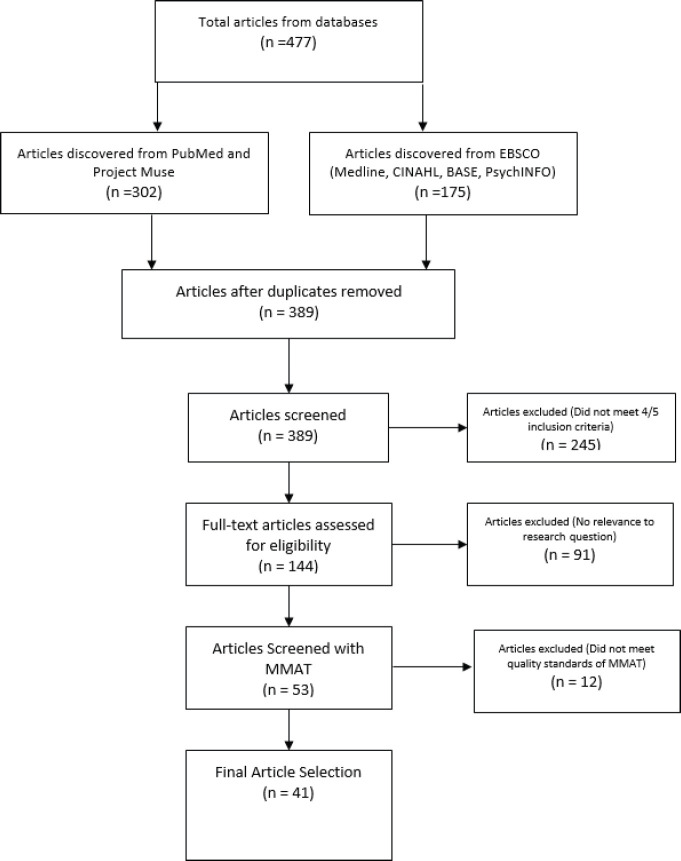
Flow diagram of data search and results.

The total number of eligible articles was 53, which were then examined with the Mixed Methods Appraisal Tool (MMAT) for quality checks [26]. This tool was chosen over others because it offered a guide to evaluate articles that used both quantitative and qualitative methods. This process showed that some articles were of low quality and others offered limited data to the research questions. The MMAT filtered the remaining 53 articles eliminating 12. The remaining articles were placed in one of two categories. The first being interventions which were articles providing some solutions to a barrier. The second was perceptual barriers which captured the attitudes and beliefs of AA, physicians and researchers. This category also housed articles discussing specific barriers. Intervention studies totalled 16, and perceptual barriers totalled 25 which led to 41 unique articles (*n* = 41). Figure 1 summarises the process of article selection.

## Results

To answer the research questions: (1) Why do AA participate in CCT at lower rates compared to other racial/ethnic groups and (2) Are there any definitive tools that have helped increase AA participation or addressed the barriers associated with their lack of participation, two categories were created (perceptual barriers and intervention studies) for the eligible article (*n* = 41). These categories were created to organise and synthesise the data being retrieved. Subsequently, the categories perceptual barriers and intervention studies were utilised to organise the articles. Perceptual barriers encompassed the physicians’, researchers’ and AAs’ feelings towards CCT as well as the actual barriers discussed by all three groups. Intervention studies were those that developed a tool with feedback from AA and also addressed a specific barrier with some sort of intervention programme. Articles exploring specific barriers associated with low AA participation were grouped in perceptual barriers to support those ideas. After analysing the articles, 25 articles addressed perceptual barriers and/or actual barriers, while 16 explored intervention strategies. Of the 16 intervention studies, seven provided evidence of an increase in AA participation or willingness after their intervention, and two studies failed to improve AA accrual numbers and/or knowledge of CCT. These categories, as well as their subcategories, are discussed below:

### Perceptual barriers

Trust, provider-related barriers, influence of family members, socioeconomic status, health literacy and spirituality were subcategories under this main category.

#### Trust

Among the 25 articles that were reviewed and categorised in the perceptual barrier category, the most common factor that impacted AA participation was trust. In multiple studies, AA appeared to have a lack of trust in the physicians caring for them [13, 17–20]. In one study carried out by Haynes-Maslow *et al* [13], eight focus groups were conducted, and the theme of trust in the medical system and physicians came up a total of 46 times. Study participants reported being concerned with physicians potentially being compensated for offering CCT. This concern eventually decreased their trust for the physician and willingness to participate in a CCT if they knew someone paid the physician to recruit them. Interestingly, some AA demonstrated more trust in their primary care physicians rather than their oncologists [13, 17, 19]. Two primary care physicians who participated in one of the focus groups detailed stories of AA patients receiving treatment options from multiple oncologists. These treatment options were then brought before their primary care physicians to receive a final word of advice due to their long-standing relationship [17]. This lack of trust for the oncologists was agreed upon by seven other healthcare providers (physicians and nurse practitioners) in the same focus group.

Some of the most frequent comments made by AA that support this idea of mistrust for the medical system were in reference to the Tuskegee trials which emerged in multiple focus group studies [13, 15, 22–24]. Participants in one focus group stated that the younger generation would most likely participate but not the older generation due to the Tuskegee syphilis study [14]. Wenzel *et al* [22] also presented data from a focus group where older AA participants stated that the Tuskegee events directly hindered their willingness to participate in CCT. This same study saw a higher average age for those who declined to participate in a CCT (57.0) as opposed to those who accepted a CCT offer (54.2). Two articles reported AA males stating their reasons for lack of trust, which included the belief that researchers only cared about money and conspiracy or hidden agendas regarding health outcomes [23, 27].

Fears of repeat ethical infractions like the Tuskegee trials were not the only cause for lack of trust for biomedical research or CCT participation among AA. A sense of fear about being used as a guinea pig was another common theme elicited in multiple studies [14, 19, 23, 28, 29] and other fears, including CCT benefitting only non-minority groups [23]. Two studies employing focus groups reported participant’s recommendation that clinical trial recruiters target everyone and not just AA because it appeared suspicious [13, 32]. AA participants in some of these studies were also concerned with information being potentially withheld from them, such as the risks of a CCT and some expressed the lack of the provision of all of the necessary information needed to make an informed decision [23, 24]. Furthermore, many articles discussed AA participants’ strong preference for CCT that did not involve the use of placebos [13, 24, 28, 29]. Moreover, on average, most participants questioned the use of control groups in research trials as they asserted this created a sense of otherness [29].

Additionally, researchers exploring the willingness to participate in a clinical trial found major differences between racial/ethnic groups. On a 5-point scale (i.e. 1 = less trust, 5 = more trust), AA had lesser trust in physicians (4.04) compared to their white counterparts (4.43) (p < 0.001) [18] and held stronger beliefs in alternative medicine (5.99) compared to whites (4.93) when measured on a 10-point scale (i.e. 1 = weaker belief, 10 = stronger belief) (*p* < 0.001) [18]. A different study looking at willingness to participate in clinical trials among AA and Whites with previous exposure to clinical trials saw contrary results. AA and Whites had no significant difference in willingness to participate nor having trust in their physicians when asked about their opinion of various types of clinical trials [57].

#### Provider-related barriers

Although lack of trust among AA in the biomedical research process appears to be a major barrier to their participation in CCT, the research process responsible for enrolling potential participants contributes significantly to the low enrollment rates. Multiple studies employing focus groups comprising researchers and physicians as participants expressed the lack of adequate information about the CCT process. In a study with healthcare providers, some physicians stated major issues with the information provided to them about CCT [17]. Specifically, when new information was provided to them to promote CCTs from their local cancer centres, there was no clear mechanism detailing the procedure to enroll a patient. Another study with physician participants also stated they needed more information and confidence about the trial before they would recommend it to a patient [19]. Physicians participating in other studies also complained about the time constraints during consultations with their patients, which directly restricted their ability to explain a clinical trial in depth [19, 32]. Supporting these findings was a study that examined the discussion trends of clinical trials among newly diagnosed lung and colorectal cancer patients [33]. In this study, 1,114 (14.1%) of those newly diagnosed with cancer reported discussing clinical trials as a potential solution. Among AA participants (*n* = 1,031), only 11.3% reported discussing a CCT when compared to their White counterparts (14.9%). Similar trends were observed in another study that reported that clinical trials were being mentioned far less in AA consultations. On average, the mention of a CCT averaged around (*M* = 2.73) compared to Whites (*M* = 4.27) [34].

Besides physician perceived barriers, another healthcare-related barrier was eligibility criteria. Two studies determining potential enrollment barriers discovered that AA were more than likely to be ineligible for a trial due to comorbidities, among other factors [36, 37]. Examples of these comorbidities as cited in published literature included hypertension, vision loss, diabetes and arthritis [30, 31]. Furthermore, another study exploring eligibility criteria in CCT found that 47.9% (192) of eligible AA men were excluded from 401 trials due to benign neutropenia (i.e. Absolute neutrophil count < 1.5 × 10 9 cells/L) even though evidence has shown that there is no increased risk of infection [38].

Serum creatinine, which is typically used as a renal function determinant, was also disproportionately used to exclude AA men. 25.2% (101) of prospective AA men were excluded due to high serum creatinine levels even though AAs are known to have, on average, higher serum creatinine levels. This was not accounted for by researchers [38].

#### Influence of family members

Studies exploring the perceptions and barriers related to AA participation have often cited family influence, socioeconomic status and health literacy as major determinants for participation. Numerous studies stated family members heavily influenced a patient’s decision-making process regarding potentially joining a trial [17, 22, 24, 28, 29, 37]. Several studies described patients being overwhelmed by family members to decline participation [22, 28, 37]. Not only were family members influential in the process, but many patients looked to family members for CCT information [22]. In some instances, patients looked to family members for information rather than the physician [29].

#### Socioeconomic status

Martinez *et al* [17] cite low socioeconomic status as a barrier to CCT participation among AA. In a study conducted using focus groups, participants expressed being fearful of the potential time and financial loss they would incur from participating in a CCT [17]. In another study, one participant directly stated that they would rather get a treatment that would ‘deal with the situation’ (cancer) than go into a CCT and deal with the financial strain that it would possibly cause [22]. Some of these financial strains were cited in various studies and they include health insurance [22, 40] and transportation [17, 25, 32, 37, 39, 40]. A study used cartographic mapping to illustrate the role of transportation in participating in CCT among AA by examining the population density of AA relative to the cancer centre’s location. Areas expected to have the highest accrual, such as those with the highest AA density, did not have higher accrual numbers of minorities, suggesting distance was a possible contributor to lack of participation [25]. AAs, from their findings, travelled the least distance (median = 5.85 miles) to a CCT compared to Whites who travelled on average further (median = 12.92 miles) [25].

#### Health literacy

One major barrier contributing to a lack of participation in CCT among AA was lower health literacy [13, 24, 28, 29]. Evans *et al* [15] prefaced health literacy as a barrier to participation in CCT among AAs because of the widespread misconceptions about placebos. Some participants in this study stated that all CCT used placebos and thus, there was a chance not to receive the much-needed treatment for cancer if needed [15]. Another misconception surrounding placebos was the idea of old and new treatments being used [15]. One study conducted among AAs reported participants complaint about the ‘fairness’ of not receiving the ‘new’ treatment, especially in CCTs using placebos. Additional findings from this study reported that AA and Hispanics were less likely to participate in a clinical trial if they were not told about being in the experimental or control group (*p* = 0.008) [18].

A general lack of understanding among AAs for the research process and medical terms such as clinical trials and medical research were demonstrated in multiple studies [13, 17, 32]. Corroborating these findings was a study determining the cancer health literacy among AA, Whites and Hispanics [41]. With a sample population (*n* = 1,500), composed equally of Whites, AA and Hispanics, AA scored the lowest (mean of 4.78) on a cancer health literacy test compared to whites (mean of 22.52) (*p* < 0.0001) [41]. This lower rate of health literacy might suggest a lack of understanding about the informed consent process in biomedical research, which also appears to be a frequent barrier to participation in CCT among AA [14, 21, 37, 39].

#### Spirituality

Spirituality has been shown to facilitate and sometimes deter AA participation in CCT. In various studies conducted via focus groups, patients explained that prayer was essential in the decision to join a CCT [13, 22]. Many AAs stated they would never join a CCT because they believed they would be healed by God [13, 22]. On the other hand, there were also AAs who would leave their health in the doctors’ hands and believed God would use the doctor as a locus of control to heal them [23]. One article looking at the correlation between spirituality and willingness to participate saw significant likelihood to participate in health-related research. For every point scored on the spirituality test, there was a 24% increase in willingness to participate (OR = 1.24, 95% CI = 1.07–1.44, *p* < 0.01) [42]. On the contrary, another study by Meng *et al* [20] examining various factors influencing willingness to participate saw no association between religious beliefs and CCT participation. Religious activity (praying), however, was positively and significantly associated with willingness to participate in CCT among AA (*β* = 0.31, *p* < 0.05) [20].

A minor barrier associated with spirituality came in the form of recruitment strategies. According to Haynes-Maslow *et al* [13], AA across all their focus groups believed researchers should recruit for CCT in churches. Despite this recommendation, principal investigators who responded to a survey by Tanner *et al* [43] indicated that they were rarely recruiting from faith-based organisations. On a 5-point scale, faith-based organisations were third to last on a list of 13, which detailed recruitment strategies of principal investigators for minorities (*M* = 1.74) [43].

### Interventions

Educational video interventions and community-based participatory research (CBPR)

#### Educational video interventions and CBPR

To address the poor accrual of AA in CCT, many researchers have looked to educational videos to educate prospective participants and increase their knowledge of CCT [44–47]. In four studies, researchers developed an educational video with three resulting in changes in knowledge and an increase in positive attitudes towards clinical trials. A pilot study by Banda *et al* [44] reported a significant increase in willingness to participate with the use of their video intervention. Prior to viewing the video, 45.4% (49 out of 108) of study participants exhibited a willingness to participate in a CCT, however, after implementing the video, the willingness to participate increased to 79.6% (86 out of 108; McNemar’s *χ*2 = 33.39, *p* < 0.001) [44]. In a similar study by Robinson *et al* [46], there was an increase in positive attitudes and willingness to participate in CCT with the use of a video intervention (52% pre-video versus 66% post-video (*p*  <  0.001). The same study saw 39/200 (19.5%) of participants agree to enroll in a CCT, and a significant change in CCT follow-ups was observed (*p*  <  0.05) as well as an overall 7.5% increase in AA enrollment [46]. However, a handful of other studies reported lesser convincing results, with one showing a small change in pre-test (73.16%) and post-test scores (76.84%) in relation to the participants’ knowledge of CCT [45] and another reporting no significant change in knowledge levels and willingness to participate in CCT [47]. Specifically, both the control and intervention groups had little to no variation in the knowledge from their analysis of variance analysis, and no difference was observed in CCT enrollment (*Z* = 0.39, *p* = 0.69) [47].

In addition to video interventions, CBPR which integrates community members in the research design was used. Four studies employed a CBPR method in different ways with varying results [48–51]. Overall, these studies showed markedly improved AA participation, with one showing a 373% increase in enrollment (22 to 104 enrollees) in 11 months [49]. A second study saw 88% of its participants consent to a health check follow-up in 2 years after they were informed about the importance of breast CCT from community-based events [48]. Two more studies using CBPR also demonstrated improvements in AA participation rate. One of them utilised community health advisors to measure adherence/retention rates. Eighty percent (251) of their intervention group followed up for a clinical trial, while only 65% of the control (131) followed up (*p* < 0.0001) [50]. CBPR was also used in the study by Green *et al* [51] which designed three modules with community leaders and investigators. Only one of the three modules, titled ‘workshop’, yielded significant increases in participants’ willingness to join a CCT or prevention trial [51].

Of the many intervention studies identified, a single study presented the idea of a patient navigator, which had a major impact on AA participation [52]. This was done by hiring lay individuals who were trained by a culturally diverse group of healthcare professionals in three didactic modules. These modules educated the trainees on the research process, the patient navigator’s job and explaining CCT to patients. In the Increasing Minority Participation Clinical Trials project conducted between 2007 and 2014, 304 patients were enrolled in a trial, with 272 (89.4%) consenting to the use of a patient navigator. It was later determined that 74.5% of those receiving a patient navigator’s guidance completed the clinical trials, whereas those without a patient navigator only saw 37.5% complete the clinical trial, which was statistically significant (*p* < 0.001).

## Discussion

The reasons for the lower participation rates among AA in CCT from the 41 articles that were eligible for review included (1) poor trust in the biomedical research system, (2) healthcare provider-related barriers, (3) familial influence, (4) socioeconomic status, (5) health literacy and (6) spirituality. In terms of solutions or tools designed to improve participation, (1) CBPR, (2) educational tools and (3) patient navigators were used.

Of the major factors contributing to the poor engagement of AA in CCT, trust appears to be the biggest issue. It is also a factor that can be easily modified with the appropriate interventions. Although AA have had general distrust in the biomedical research process for quite some time [53], very little in terms of solutions have been proposed. The most common proposal for solving this issue has been increasing diversity in the healthcare workforce. Studies have shown that patients who are racially concordant with their physicians are more likely to be receptive to medical advice [60–62]. Not only is adherence to physician medical advice improved but also patient–physician communication [63, 64], patient perceptions of treatments [62, 64] and even a patient’s understanding of their risk for cancer [65]. One study has even implicated physician–newborn race concordance with a mortality rate that is halved in AA babies when they are cared for by AA physicians [54]. Organisations such as the NIH have committed to diversifying the biomedical and healthcare workforce with the use of grants and various programmes that target underrepresented minorities. Although this has proven helpful [66], significant changes to the physician workforce will only be seen years from now as it takes a long time to recruit and train physicians and other biomedical professionals from underrepresented minority communities.

Fast and effective means to increasing CCT participation among AA can possibly be ameliorated with the use of AA nurses or primary care physicians who are far more accessible than AA oncologists, who make up less than 5% of all oncologists [58]. Above and beyond their race, these healthcare providers should be trained in cultural competency as well as be more integrated with oncology treatment teams and should be involved in the oncology consultations for which CCT are offered. Their duties can involve sharing clinical trial information, educating patients/families and acting as a liaison between the patient and research team to improve trust. This will be an effective use of nurses and gatekeepers such as primary care providers, given the limited time oncologist spend with patients [19, 32].

Another potential solution involves training nurses to conduct small research tasks that are needed for the production of experimental drug or medical tool. From these tasks and projects, educational videos can be produced portraying the AA nurses actively engaged in the biomedical research process, a domain typically dominated by White males. As previously mentioned, three of four studies using video interventions successfully improved AA perceptions and/or participation in CCT. These interventions can be improved by involving the more available AA nurses in the videos as they are engaged in the research process. This will benefit both the potential CCT participants and nurses in a variety of ways. For one, nursing as a profession has been known to be stressful, which is evident from the turnover rate [67, 68]. By allowing nurses to transfer to a wet lab during a shift can offer some relief from the hectic hospital environment. In addition, allowing the nurses to experience what working in a lab is like can also stir up some interest in the biomedical field which has been a goal of the NIH for quite some time [66]. As of 2014, 17.5% of all nursing graduates leave their first job within the first year and one in three leaves within 2 years [56]. These graduates could be recruited instead of the active nurses, thus preventing further reduction to the nurse workforce.

Furthermore, efforts to improve participation among AA should utilise a CBPR strategy that fosters co-learning and capacity building among researchers and the community. This will enable community members to provide input and knowledge about the community’s perception of CCT, impediments to recruiting participants and recommendations to overcome existing barrier to participation in CCT [55]. Such input would benefit researchers in designing strategies aimed at addressing specific barriers identified by community members. Employing a CBPR approach also establishes a form of trust between researchers and the community which is an important barrier to participation in CCT among AA [59]. Faith-based organisations, churches and mosques are trusted institutions in many AA communities and provide opportunity for recruiting community members in research efforts. By engaging, educating and recruiting leaders from these institutions such as pastors, elders and imams can foster bonds of trust among congregants and the research team responsible for CCT. Building on the strengths and resources within a community while engaging in collaborative research and forming equitable partnership has the potential to improve community buy in and participation in CCT [55].

Besides trust and healthcare provider-related behaviours, socioeconomic status, family influence, health literacy and spirituality all appear to be a result of sociological factors with no clear instantaneous solution. Lack of trust in biomedical research appears to be an area that if remedied, can offer the single largest jump in AAs participation in CCT. Further studies are needed to determine the best plan of action to address these barriers. Finally, future interventions should consider incorporating culturally competent training for their biomedical research and healthcare team, especially in areas with limited cultural diversity.

## Study limitations

While relevant databases were searched, it is possible that some articles may have been left out during this process. Publication bias is also another limitation. It is likely that the articles included in our analyses were those with significant findings.

## Conclusion

The results of this study confirm previous barriers and reasons for the low participation rates of AA participation in CCT noted in published literature. Although these barriers are numerous, the solutions are not. Once the sociological factors impacting CCT barriers are weighed, the solutions to mitigating these issues can be drawn from the very same biomedical research institutions that offer CCT opportunities. Individuals educating the patients on CCT, whether it be a physician, nurse or research coordinator, should be briefed on preconceived notions and misunderstandings common in AA communities regarding CCT. This will enable the researcher to clarify misperceptions, address questions or concerns that potential participants may have about CCT and provide them with the necessary information to make informed decisions about participation.

The most important solution would be increasing the diversity of the biomedical research and healthcare team that is involved in offering the clinical trials. Integrating culturally competent nurses into oncology treatment teams has the potential to improve interaction and communication with patients given the time constraints that oncologists face. Nurses could spend more time educating patients, particularly those with a knowledge deficit about the conduct of clinical trials. Enhancing the level of communication and education of cancer patients from minority communities will build trust between the patient, oncology team and has the potential to improve participation rates in clinical trials among this population. When the patient has no trust, and little connection to the individuals treating them, it might be difficult educating them on the importance of participating in CCT.

## List of abbreviations

NIH, National Institutes of Health; AA, African American; CDC, Centers for Disease Control and Prevention; CCTs, Cancer clinical trials; CBPR, Community based participatory research; MMAT, Mixed Methods Appraisal Tool.

## Conflicts of interest

There are no conflicts of interest.

## Author contributions

Jordan Swaby carried out all the stages of the systematic review under the guidance of Dr Kaninjing and Dr Motolani Ogunsanya.

## Funding

This research was funded in whole by the National Cancer Institute (NCI) R25CA21422: The National Institute of Health (NIH) U54CA233444 (NIH): & supported by the University of Florida’s ReTOOL Program 1R25CA214225-01

## References

[1] Center for Drug Evaluation and Research (2020). Enhancing the diversity of clinical trial populations - eligibility criteria, enrollment practices, and trial designs guidance for industry. https://www.fda.gov/regulatory-information/search-fda-guidance-documents/enhancing-diversity-clinical-trial-populations-eligibility-criteria-enrollment-practices-and-trial.

[2] National Institutes of Health NIH policy and guidelines on the inclusion of women and minorities as subjects in clinical research. https://grants.nih.gov/policy/inclusion/women-and-minorities/guidelines.htm.

[3] Centers for Disease Control and Prevention (2020). Health disparities among youth. https://www.cdc.gov/healthyyouth/disparities/index.htm.

[4] Jemal A, Thun MJ, Ries LA (2008). Annual report to the nation on the status of cancer, 1975-2005, featuring trends in lung cancer, tobacco use, and tobacco control. J Natl Cancer Inst.

[5] DeSantis CE, Fedewa SA, Goding Sauer A (2016). Breast cancer statistics, 2015: convergence of incidence rates between black and white women. CA Cancer J Clin.

[6] National Cancer Institute African American disparities in low-grade prostate cancer death. https://www.cancer.gov/news-events/cancer-currents-blog/2019/prostate-cancer-death-disparities-black-men.

[7] Tsodikov A, Gulati R, de Carvalho TM (2017). Is prostate cancer different in black men? Answers from 3 natural history models. Cancer.

[8] U.S. Cancer Statistics Working Group U.S. cancer statistics data visualizations tool. www.cdc.gov/cancer/dataviz.

[9] Newman LA (2017). Breast cancer disparities: socioeconomic factors versus biology. Ann Surg Oncol.

[10] Street RL, Makoul G, Arora NK (2009). How does communication heal? Pathways linking clinician-patient communication to health outcomes. Patient Educ Couns.

[11] Ahaghotu C, Tyler R, Sartor O (2016). African American participation in oncology clinical trials--focus on prostate cancer: implications, barriers, and potential solutions. Clin Genitourin Cancer.

[12] U.S Census Bureau U.S. Census Bureau quickfacts: United States Census Bureau QuickFacts. www.census.gov/quickfacts/fact/table/US/PST045219.

[13] Haynes-Maslow L, Godley P, Dimartino L (2014). African American women’s perceptions of cancer clinical trials. Cancer Med.

[14] Owens OL, Jackson DD, Thomas TL (2013). African American men’s and women’s perceptions of clinical trials research: focusing on prostate cancer among a high-risk population in the South. J Health Care Poor Underserved.

[15] Evans KR, Lewis MJ, Hudson SV (2012). The role of health literacy on African American and Hispanic/Latino perspectives on cancer clinical trials. J Cancer Educ.

[16] Commissioner O Diversity in clinical trial participation. https://www.fda.gov/patients/clinical-trials-what-patients-need-know/diversity-clinical-trial-participation.

[17] Sprague Martinez L, Freeman ER, Winkfield KM (2017). Perceptions of cancer care and clinical trials in the black community: implications for care coordination between oncology and primary care teams. Oncologist.

[18] Pariera KL, Murphy ST, Meng J (2017). Exploring willingness to participate in clinical trials by ethnicity. J Racial Ethn Health Disparities.

[19] Robinson BN, Newman AF, Wallington SF (2016). Focus on you: cancer clinical trials perspectives. Contemp Clin Trials Commun.

[20] Meng J, McLaughlin M, Pariera K (2016). A comparison between caucasians and African Americans in willingness to participate in cancer clinical trials: the roles of knowledge, distrust, information sources, and religiosity. J Health Commun.

[21] Sadler GR, Gonzalez J, Mumman M (2010). Adapting a program to inform African American and Hispanic American women about cancer clinical trials. J Cancer Educ.

[22] Wenzel JA, Mbah O, Xu J (2015). A model of cancer clinical trial decision-making informed by African-American cancer patients. J Racial Ethn Health Disparities.

[23] Somayaji D, Cloyes KG (2015). Cancer fear and fatalism: how African American participants construct the role of research subject in relation to clinical cancer research. Cancer Nurs.

[24] Rivers DA, Pal T, Vadaparampil ST (2019). A community-academic partnership to explore informational needs of African American women as a primer for cancer clinical trial recruitment. Ethn Health.

[25] Bruner DW, Pugh SL, Yeager KA (2015). Cartographic mapping and travel burden to assess and develop strategies to improve minority access to national cancer clinical trials. Int J Radiat Oncol Biol Phys.

[26] McGill University Department of Family Medicine (2018). Mixed methods appraisal tool.

[27] U.S. Food and Drug Administration (2011). Dialogues on diversifying clinical trials: successful strategies. https://www.fda.gov/science-research/womens-health-research/dialogues-diversifying-clinical-trials.

[28] Brown RF, Cadet DL, Houlihan RH (2013). Perceptions of participation in a phase I, II, or III clinical trial among African American patients with cancer: what do refusers say?. J Oncol Pract.

[29] Schapira MM, Mackenzie ER, Lam R (2014). Breast cancer survivors willingness to participate in an acupuncture clinical trial: a qualitative study. Support Care Cancer.

[30] Unger JM, Hershman DL, Fleury ME (2019). Association of patient comorbid conditions with cancer clinical trial participation. JAMA Oncol.

[31] Fowler H, Belot A, Ellis L (2020). Comorbidity prevalence among cancer patients: a population-based cohort study of four cancers. BMC Cancer.

[32] Davis TC, Arnold CL, Mills G (2019). A qualitative study exploring barriers and facilitators of enrolling underrepresented populations in clinical trials and biobanking. Front Cell Dev Biol.

[33] Kehl KL, Arora NK, Schrag D (2014). Discussions about clinical trials among patients with newly diagnosed lung and colorectal cancer. J Natl Cancer Inst.

[34] Eggly S, Barton E, Winckles A (2015). A disparity of words: racial differences in oncologist-patient communication about clinical trials. Health Expect.

[35] NIH Cancer disparities. https://www.cancer.gov/about-cancer/understanding/disparities.

[36] Langford AT, Resnicow K, Dimond EP (2014). Racial/ethnic differences in clinical trial enrollment, refusal rates, ineligibility, and reasons for decline among patients at sites in the National Cancer Institute's Community Cancer Centers Program. Cancer.

[37] Penberthy L, Brown R, Wilson-Genderson M (2012). Barriers to therapeutic clinical trials enrollment: differences between African-American and white cancer patients identified at the time of eligibility assessment. Clin Trials.

[38] Vastola ME, Yang DD, Muralidhar V (2018). Laboratory eligibility criteria as potential barriers to participation by black men in prostate cancer clinical trials. JAMA Oncol.

[39] Jackson DD, Owens OL, Friedman DB (2014). An intergenerational approach to prostate cancer education: findings from a pilot project in the southeastern USA. J Cancer Educ.

[40] Muñoz-Antonia T, Ung D, Montiel-Ishino FA (2015). African Americans' and Hispanics' information needs about cancer care. J Cancer Educ.

[41] Echeverri M, Anderson D, Nápoles AM (2018). Cancer health literacy and willingness to participate in cancer research and donate bio-specimens. Int J Environ Res Public Health.

[42] Ojukwu E, Powell LR, Person SD (2018). Spirituality and willingness to participate in health-related research among African Americans. J Health Care Poor Underserved.

[43] Tanner A, Kim SH, Friedman DB (2015). Promoting clinical research to medically underserved communities: current practices and perceptions about clinical trial recruiting strategies. Contemp Clin Trials.

[44] Banda DR, Libin AV, Wang H (2012). A pilot study of a culturally targeted video intervention to increase participation of African American patients in cancer clinical trials. Oncologist.

[45] Pelto DJ, Sadler GR, Njoku O (2016). Adaptation of a cancer clinical trials education program for African American and Latina/o community members. Health Educ Behav.

[46] Robinson BN, Newman AF, Tefera E (2017). Video intervention increases participation of black breast cancer patients in therapeutic trials. NPJ Breast Cancer.

[47] Skinner JS, Fair AM, Holman AS (2019). The impact of an educational video on clinical trial enrollment and knowledge in ethnic minorities: a randomized control trial. Front Public Health.

[48] Smith A, Vidal GA, Pritchard E (2018). Sistas taking a stand for breast cancer research (STAR) study: a community-based participatory genetic research study to enhance participation and breast cancer equity among African American women in Memphis, TN. Int J Environ Res Public Health.

[49] Germino BB, Mishel MH, Alexander GR (2011). Engaging African American breast cancer survivors in an intervention trial: culture, responsiveness and community. J Cancer Surviv.

[50] Fouad MN, Johnson RE, Nagy MC (2014). Adherence and retention in clinical trials: a community-based approach. Cancer.

[51] Green MA, Michaels M, Blakeney N (2015). Evaluating a community-partnered cancer clinical trials pilot intervention with African American communities. J Cancer Educ.

[52] Fouad MN, Acemgil A, Bae S (2016). Patient navigation as a model to increase participation of African Americans in cancer clinical trials. J Oncol Pract.

[53] Armstrong K, Ravenell KL, McMurphy S (2007). Racial/ethnic differences in physician distrust in the United States. Am J Public Health.

[54] Greenwood BN, Hardeman RR, Huang L (2020). Physician-patient racial concordance and disparities in birthing mortality for newborns. Proc Natl Acad Sci U S A.

[55] Israel BA, Schulz AJ, Parker EA (2001). Community-based participatory research: policy recommendations for promoting a partnership approach in health research. Educ Health (Abingdon).

[56] Kovner CT, Brewer CS, Fatehi F (2014). What does nurse turnover rate mean and what is the rate?. Policy Polit Nurs Pract.

[57] Durant RW, Legedza AT, Marcantonio ER (2011). Willingness to participate in clinical trials among African Americans and whites previously exposed to clinical research. J Cult Divers.

[58] Hamel LM, Chapman R, Malloy M (2015). Critical shortage of African American medical oncologists in the United States. J Clin Oncol.

[59] Holkup PA, Tripp-Reimer T, Salois EM (2004). Community-based participatory research: an approach to intervention research with a native American community. ANS Adv Nurs Sci.

[60] Strumpf EC (2011). Racial/ethnic disparities in primary care: the role of physician-patient concordance. Med Care.

[61] Traylor AH, Schmittdiel JA, Uratsu CS (2010). Adherence to cardiovascular disease medications: does patient-provider race/ethnicity and language concordance matter?. J Gen Intern Med.

[62] Saha S, Beach MC (2020). Impact of physician race on patient decision-making and ratings of physicians: a randomized experiment using video vignettes. J Gen Intern Med.

[63] Cooper-Patrick L, Gallo JJ, Gonzales JJ (1999). Race, gender, and partnership in the patient-physician relationship. JAMA.

[64] Penner LA, Dovidio JF, Gonzalez R (2016). The effects of oncologist implicit racial bias in racially discordant oncology interactions. J Clin Oncol.

[65] Persky S, Kaphingst KA, Allen VC (2013). Effects of patient-provider race concordance and smoking status on lung cancer risk perception accuracy among African-Americans. Ann Behav Med.

[66] Valantine HA, Lund PK, Gammie AE (2016). From the NIH: a systems approach to increasing the diversity of the biomedical research workforce. CBE Life Sci Educ.

[67] Halter M, Pelone F, Boiko O (2017). Interventions to Reduce Adult Nursing Turnover: A Systematic Review of Systematic Reviews. Open Nurs J.

[68] Flinkman M, Isopahkala-Bouret U, Salanterä S (2013). Young registered nurses’ intention to leave the profession and professional turnover in early career: a qualitative case study. ISRN Nurs.

